# The Structures and Binding Modes of Small-Molecule Inhibitors of *Pseudomonas aeruginosa* Elastase LasB

**DOI:** 10.3390/antibiotics11081060

**Published:** 2022-08-04

**Authors:** Virgyl Camberlein, Gwenaëlle Jézéquel, Jörg Haupenthal, Anna K. H. Hirsch

**Affiliations:** 1 Helmholtz Institute for Pharmaceutical Research Saarland (HIPS)—Helmholtz Centre for Infection Research (HZI), Campus E8.1, 66123 Saarbrücken, Germany; 2 Department of Pharmacy, Saarland University, Campus E8.1, 66123 Saarbrücken, Germany

**Keywords:** antivirulence therapy, antimicrobial resistance, small-molecule inhibitors, *Pseudomonas aeruginosa* infection, elastase LasB

## Abstract

Elastase B (LasB) is a zinc metalloprotease and a crucial virulence factor of *Pseudomonas aeruginosa*. As the need for new strategies to fight antimicrobial resistance (AMR) constantly rises, this protein has become a key target in the development of novel antivirulence agents. The extensive knowledge of the structure of its active site, containing two subpockets and a zinc atom, led to various structure-based medicinal chemistry programs and the optimization of several chemical classes of inhibitors. This review provides a brief reminder of the structure of the active site and a summary of the disclosed *P. aeruginosa* LasB inhibitors. We specifically focused on the analysis of their binding modes with a detailed representation of them, hence giving an overview of the strategies aiming at targeting LasB by small molecules.

## 1. Introduction

Opportunistic Gram-negative bacteria such as *Pseudomonas aeruginosa* particularly harm immunocompromised patients. Thus, *P. aeruginosa* infections represent a major cause of nosocomial infections and are the main cause of mortality in cystic fibrosis patients [1]. Endowed with remarkable mechanisms of adaptation, survival and resistance to antibiotics, which make it difficult to eliminate, this bacterium is classified as a critical pathogen by the World Health Organization (WHO) [2]. According to the European Centre for Disease Prevention and Control (ECDC), 18% of the *P. aeruginosa* isolates have demonstrated resistance to two or more antimicrobial classes in Europe in 2019 [3]. In the USA, a report from the Centers for Disease Control and Prevention (CDC) estimated that multidrug-resistant *P. aeruginosa* strains have affected 32,600 hospitalized patients and killed 2700 in 2017, with an associated health cost of USD 767M [4]. The spread of multidrug-resistant *P. aeruginosa* strains continues to increase at an alarming rate, hence the urgent necessity to develop new treatment options. The development of *P. aeruginosa* resistance is mainly due to a broad repertoire of virulence factors, which is favored by the large genome of the pathogen [5]. Those virulence factors can be either intra- or extra-cellular and fulfill different functions. The outer-membrane proteins, for example, can be involved in the maintenance of cell integrity, the diffusion channels, the adhesion or the transport of molecules [6]. Biofilm formation or flagella are other strategies used by the pathogen to escape from antibiotics [7,8,9]. Bacterial proteases are also important in the virulence process due to their ability to hydrolyze many different substrates [10,11].

In recent years, the practice of targeting bacterial virulence factors has emerged as a promising strategy to combat bacterial infections, as they significantly contribute to the pathogenicity of the bacteria [12,13,14,15,16]. For instance, the beneficial effects of targeting virulence factors were observed in *Neisseria* species [17], such as the Type IV pili inhibition by trifluoperazine that reduces the host colonization and the formation of vascular lesions [18]. Given their key role in host colonization and their extracellular localization, the bacterial collagenases are considered to be crucial virulence factors [19] and therefore represent suitable drug targets for developing antivirulence compounds [20,21,22,23]. Inhibiting *Staphylococcus aureus* virulence factors also demonstrated attractive effects [24], such as the inhibition of the *agr*-mediated quorum sensing system by savirin, which promotes the host defense [25]. The inhibition of the *P. aeruginosa* quorum sensing was also reported with promising effects on biofilm development, virulence factor secretion and host colonization [26]. Among the *P. aeruginosa* virulence factors, Elastase B (LasB) is considered to be one of the most important [27,28]; its inhibition by small molecules improves the survival rate in mice and *Galleria mellonella* larvae [29,30,31,32].

LasB is an extracellular secreted metalloprotease (Zn^2+^/Ca^2+^ cofactors) and is structurally similar to thermolysin; hence, it belongs to the M4 metalloprotease family. Firstly, this enzyme has the capacity to cleave a large diversity of host substrates such as structural (elastin, collagens, laminin, etc.) and immune system components (cytokines, immunoglobulins, etc.), promoting host invasion and immune evasion. Secondly, it is involved in the activation of bacterial-derived proteins such as flagellin, exotoxins and other proteases essential for pathogenicity. Strikingly, targeting LasB offers three advantages: reduction in selective pressure, which leads to the emergence of resistant strains, preservation of commensal bacteria, and its extracellular localization that does not require penetration of the cell wall, making this protein an attractive drug target [28,33].

## 2. Description of the Active Site of LasB

**Figure 1:**
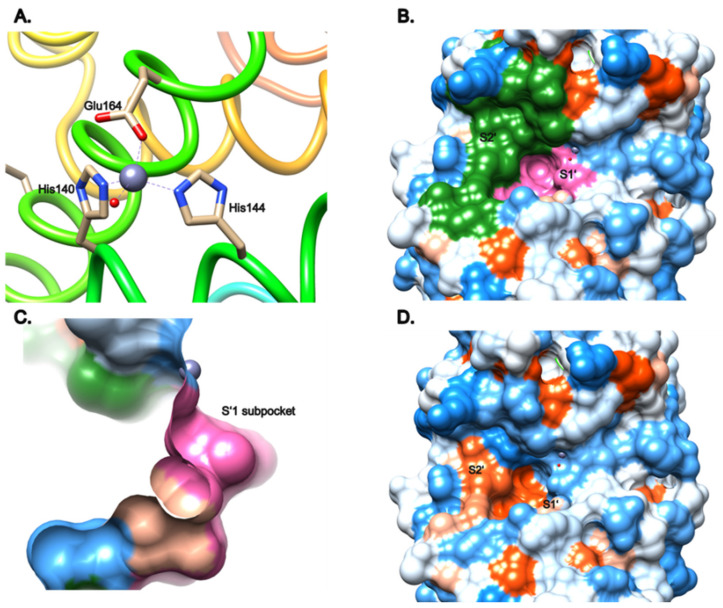
Structural description of the active site of LasB (PDB entry: 1EZM) [34] visualized using Chimera 1.15. (**A**) Tetrahedral coordination of the zinc cation by His140, His144 and Glu164, as well as a water molecule. (**B**) Visualization of the pockets S’1 (pink) and S’2 (green) surfaces. (**C**) Sideview of the deep subpocket of S’1. (**D**) Hydrophobicity of the active-site surface from blue (hydrophilic) to red (hydrophobic). These images were generated using Chimera 1.15.

Thayer et al. solved the three-dimensional structure of LasB in 1991 [34], (PDB entry: 1EZM), and described the active site as the span of two helices that provide ligands for the zinc, as well as the loop connecting them. The three ligands binding the metal are His140, His144 and Glu164, and the zinc usually adopts a tetrahedral conformation through an additional interaction with either a water molecule or the ligand (Figure 1A).

The active site consists of two pockets, S1′ and S2′, highlighted in pink and green, respectively (Figure 1B). The pocket S1′ can be described as the main interacting pocket, as it contains the zinc as well as its three ligands. It features an interesting subpocket (Figure 1C) that is both narrow and deep inside the protein, and can host small and specific moieties, as will be described later in this review. The S1′ subpocket is lipophilic, due to the aliphatic residues Leu132, Ile190, Ile186 that line it; however, the more external part of the pocket, especially close to the zinc cation, is more hydrophilic (Figure 1D). On the other hand, the S2′ pocket is more external and wider than the S1′ pocket, with a hydrophobic part in the middle, and the upper part being more hydrophilic.

The binding site of LasB thus presents various features, which have been extensively described in the literature [35], and can help rationalize the activity and inspire the design of LasB inhibitors.

## 3. LasB Inhibitors

The main strategy to inhibit LasB consists of targeting the zinc cation in the active site, through a zinc-binding group (ZBG), which allows the neutralization of the catalytic activity. Various ZBGs are used for this purpose and will be presented below. 

### 3.1. Phosphoramidate Compounds

**Figure 2:**
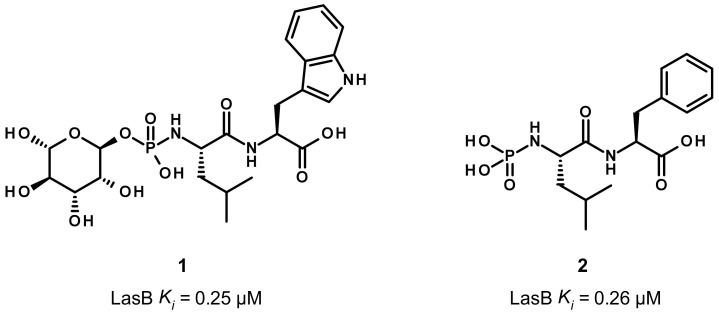
Chemical structures and biochemical activities of phosphoramidon **1** and its analogue **2**. *K_i_* values were extracted from the original publication [36].

Phosphoramidon (**1**) and its analogue **2** are two dipeptides, which contain a phosphoramidate group as ZBG (Figure 2). As the first disclosed LasB inhibitor, phosphoramidon was isolated from *Streptomyces tanashiensis* and initially reported as a thermolysin inhibitor (*K_i_* of 30 nM at neutral pH). As thermolysin and LasB share a high percentage of homology, it also showed an inhibitory activity on LasB with a *K_i_* value of 0.25 μM [36].

**Figure 3:**
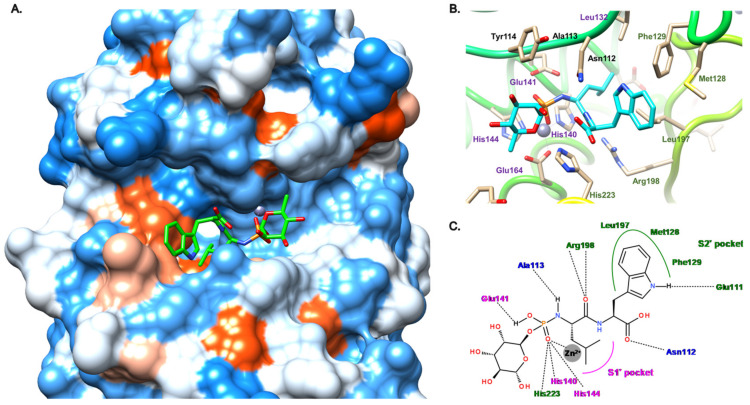
Binding mode of phosphoramidon (**1**) from PDB entry 3DBK. (**A**) View of the active site of LasB in surface mode accommodating phosphoramidon (green) visualized using Chimera 1.15. The zinc cation is represented as a gray sphere. (**B**) View of the active site of LasB with phosphoramidon (blue) and key residues (gray sticks) involved in the binding visualized using Chimera 1.15. (**C**) Two-dimensional schematic representation of the binding mode. The key interactions are indicated as dashes, the zinc cation is represented as a gray sphere, and the key residues are indicated in pink for those belonging to the S1′ pocket, green for those belonging to the S2′ pocket and blue for the others.

Regarding its binding mode (Figure 3), the phosphoryl moiety coordinates the zinc cation as expected, and interacts with three histidine residues (His140, His144, His223) through hydrogen bonds (Figure 3B,C). Additional hydrogen bonds are involved in the binding such as the hydroxyl and the NH of the phosphoramidate with Glu141 and Ala113, respectively, the carbonyl of the carboxylic acid with Asn112 and, in particular, the carbonyl of the amide group of **1** and Arg198 (Figure 3B,C). The tryptophan moiety is accommodated the S2′ pocket, which is lined by hydrophobic residues (Met128, Phe129, Leu197) and the isobutyl chain partially occupies a small hydrophobic cavity of the S1′ pocket (Figure 3). Later, the deletion of the carbohydrate moiety and the tryptophan replacement by phenylalanine led to compound **2**, which displays a similar activity as **1** (*K_i_* = 0.26 μM), thus demonstrating that the carbohydrate group and the hydrogen bond between the NH of the indole and the glutamate residue Glu111 are not essential for the activity [36].

### 3.2. Thiol Compounds

#### 3.2.1. Peptides

**Figure 4:**
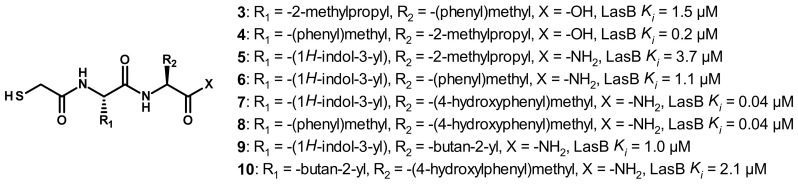
Chemical structures and biochemical activities of the thiols **3**–**10**. *K_i_* values were extracted from the original publications [35,37,40].

Following the discovery of compound **2**, Kessler et al. continued their exploration of the peptide class and replaced the phosphoramidate with a thiol as ZBG [37]; the chemical structures are indicated on Figure 4. This replacement led to a decrease in activity (compound **3**, *K_i_* = 1.5 μM vs. compound **2**, *K_i_* = 0.26 μM). However, the inversion of the side chains interestingly restores the activity and results in the equipotent inhibitor as compound **2** (compound **4**, *K_i_* = 0.2 μM). Compared to compound **3**, its D-phenylalanine analogue displays a 23-fold lower activity (*K_i_* = 34 μM) [37]. This initial work was continued by Cathcart’s group, which has explored the S1′ and S2′ pockets using a focused library of *N*-alpha mercaptoamide-containing dipeptides; the 20 naturally occurring aminoacids were successively introduced at the P’_1_ (R^1^ group) and P’_2_ (R^2^ group) positions [38,39]. The chemical formulas of six of these analogues are reported in Figure 4 (compound **5**–**10**). The best disclosed inhibitors have a tryptophan or a phenylalanine in P’_1_ position and a tyrosine in P’_2_ position (compound **7** and **8**, *K*_i_ = 0.04 μM). As a comparison, the tyrosine replacement by a phenylalanine led to a 27-fold drop in activity (compound **6**, *K_i_* = 1.1 μM vs. compound **7**, *K_i_* = 0.04 μM), demonstrating that the phenol is crucial to reach a high potency. Nonaromatic aminoacids can also be tolerated in P’_1_ and P’_2_ positions, but to a lesser extent (compound **5**, *K_i_* = 3.7 μM, compounds **9** and **10**, *K_i_* = 1.0 and 2.1 μM, respectively).

**Figure 5:**
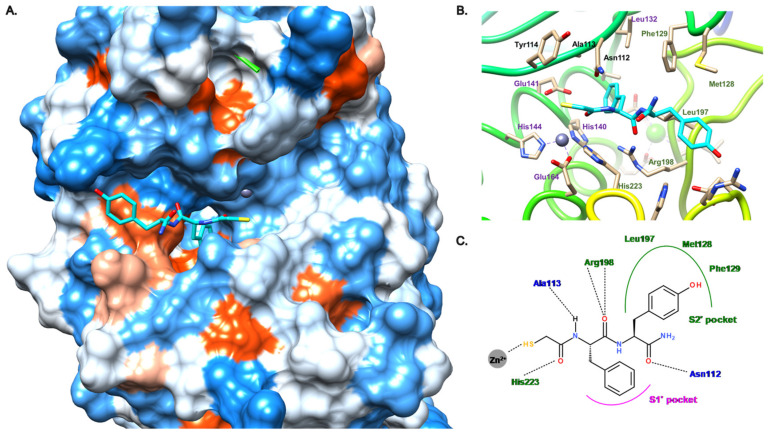
Docking simulation of compound **8** from PDB entry 3DBK using Chimera 1.15. (**A**) View of the active site of LasB, in surface mode, accommodating **8** (green) visualized using Chimera 1.15. The zinc cation is represented as a gray sphere. (**B**) View of the active site of LasB with **8** (blue) and key residues (gray sticks) involved in the binding visualized using Chimera 1.15. (**C**) Two-dimensional schematic representation of the hypothetic binding mode. The key interactions are indicated as dashes, the zinc cation is represented as a gray sphere, and the key residues are indicated in pink for those belonging to the S1′ pocket, green for those belonging to the S2′ pocket and blue for the others.

As no crystal structures with LasB are available for this family of thiols, we performed docking studies to unravel their binding mode (Figure 5). Studies on compound **8** showed that the S1′ pocket seems to accommodate the benzyl group, whereas the phenol ring lies in the S2′ pocket (Figure 5A,C). Key hydrogen bonds, the same ones involved in the binding of phosphoramidon (**1**), may be found here: (i) the carbonyl oxygen atom with Arg198, (ii) the amide nitrogen atom with Ala113 and (iii) the C-terminal carbonyl oxygen atom with Asn112 (Figure 5B,C). However, despite a potentially similar binding as phosphoramidon (**1**), the phenol moiety of **8** seems to be too far from Glu111 to establish a hydrogen bond. Our simulations are similar to those previously published for these dipeptides [35].

A tripeptide HS-CH_2_-(*DL*)Leu-Phe-Ala-NH_2_, known as a potent inhibitor of mammalian collagenases, also displays a potent activity on LasB with a *K_i_* of 0.3 μM, indicating that the peptide length extension does not significantly impact the activity [40]. Thus, these studies allowed the understanding of the mapping of the S1′ and S2′ pockets: the S1′ subsite displays a preference for bulky aromatic side chains, whereas the S2′ pocket can accept a wider scope of residues (Ile, Phe, Leu, Val, Gln, Arg, Lys, Asp, Thr and Tyr), but with a strong preference for bulky hydrophobic side chains. Before these dipeptides can be considered as possible future therapeutics, further studies are needed, for example, on selectivity and toxicity. 

#### 3.2.2. Nonpeptidic Small-Molecule Inhibitors

**Figure 6:**
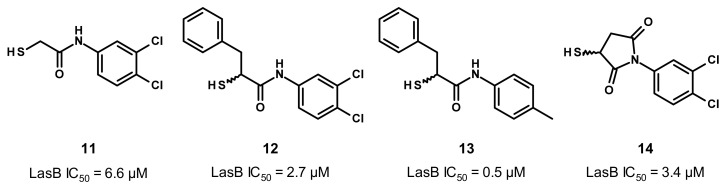
Chemical structures and inhibitory activities of the thiols **11**–**14**. IC_50_ values were extracted from the original publications [21,30,31].

As described in Figure 6, our group reported two new families of thiol inhibitors, which are articulated around *N*-aryl mercaptoacetamide (compounds **11**–**13**) and *N*-aryl mercaptosuccinimide cores (compound **14**). The *N*-aryl mercaptoacetamide family was discovered through a functional screening based on the FRET-based in vitro assay, which was previously reported by Nishino et al. [41], with a focused protease-inhibitor library [30]. This scaffold has also been reported by Janda et al. from in silico and in vitro studies [42]. Structure–activity relationship (SAR) studies led to compound **11**, which displays a moderate activity on LasB with an IC_50_ value of 6.6 μM. 

**Figure 7:**
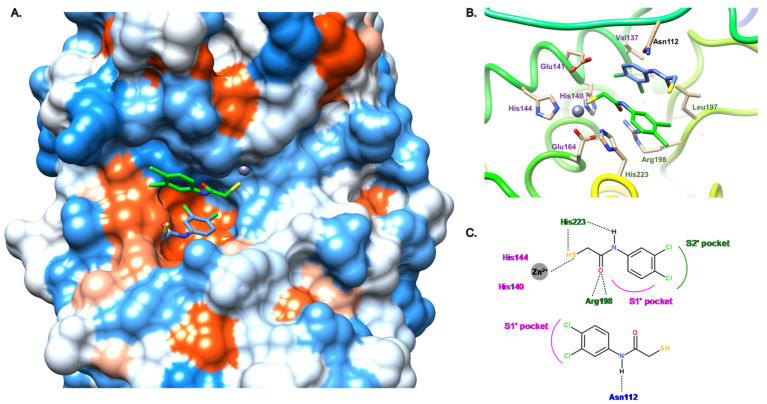
Binding mode of **11** from PDB entry 6F8B [30]. (**A**) View of the active site of LasB, in surface mode, accommodating **11a** (green) and **11b** (dark blue) visualized using Chimera 1.15. The zinc cation is represented as a gray sphere. (**B**) View of the active site of LasB with **11a** (green), **11b** (dark blue) and key residues (gray sticks) involved in the binding visualized using Chimera 1.15. (**C**) Two-dimensional schematic representation of the binding mode of **11a** (top) and **11b** (bottom). The key interactions are indicated as dashes, the zinc cation is represented as a gray sphere, and the key residues are indicated in pink for those belonging to the S1′ pocket, green for those belonging to the S2′ pocket and blue for the others.

The co-crystal structure of **11** with LasB allowed us to understand the binding mode of this compound (Figure 7). Surprisingly, two antiparallel molecules of **11** were found in the active site. For the “expected” molecule (**11a**, in green on Figure 7A,B), the thiol coordinates the zinc cation and also establishes a hydrogen bond with His223. This amino acid also interacts with the amide nitrogen atom of **11** through a hydrogen bond. Regarding the occupation of the S1′ and S2′ pockets of the active site, the aromatic core seems to occupy the S1′ subsite (with the phenyl) and partially S2′ (with the di-chloro motif). A strong bidendate hydrogen bond can be found between the amide carbonyl oxygen atom of **11** and Arg198; the S2′ subsite is not occupied enough by any part of **11** that could explain the moderate activity of the latter. For the “unexpected” molecule (**11b**, in blue in Figure 7A,B), the thiol does not coordinate the zinc cation. It binds the active site by a hydrogen bond between the amide nitrogen atom of **11** and Asn112, whereas the dichlorophenyl moiety is located in the S1′ pocket. 

**Figure 8:**
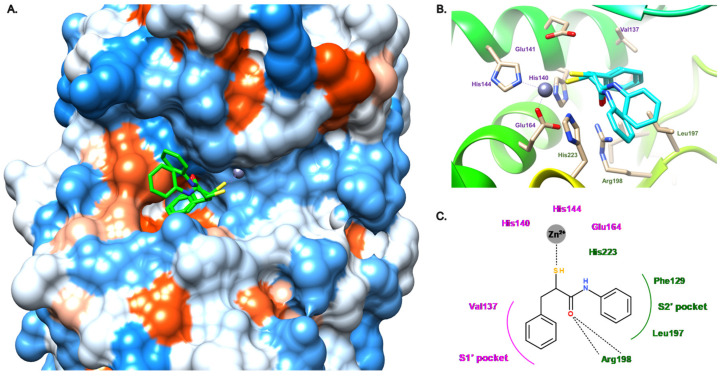
Binding mode of the dehalogenated analogue of **12** from PDB entry 7OC7 [31]. (**A**) View of the active site of LasB, in surface mode, accommodating the dehalogenated analogue of **12** (green, two conformations) visualized using Chimera 1.15. The zinc cation is represented as a gray sphere. (**B**) View of the active site of LasB with the dehalogenated analogue of **12** (blue) and key residues (gray sticks) involved in the binding visualized using Chimera 1.15. (**C**) Two-dimensional schematic representation of the binding mode of the dehalogenated analog of **12**. The key interactions are indicated as dashes, the zinc cation is represented as a gray sphere, and the key residues are indicated in pink for those belonging to the S1′ pocket and green for those belonging to the S2′ pocket.

In order to target both S1′ and S2′ subsites, we attempted to merge both molecules **11a** and **11b** through the use of *N*-aryl and *N*-aliphatic analogs. Unfortunately, this modulation did not allow us to improve the activity of **11** as these new derivatives display IC_50_ values in the two-digit micromolar range [30]. However, interestingly, the introduction of the benzyl moiety in the α-position of the amide led to a slight increase in activity (compound **12**, IC_50_ = 2.7 μM), and strikingly, improved it 13-fold when the 3,4-dichloro group was replaced by a 4-methyl motif (compound **13**, IC_50_ = 0.5 μM) [31]. This gain in activity is explained by the fact that the S1′and S2′ pocket occupancy is crucial for a high potency of the inhibitors. As indicated in Figure 8, the enhanced occupation is mainly due to the *N*-aryl moiety fitting in the S2′ pocket and being stabilized by hydrophobic interactions, in particular, with Leu197, and to the α-benzyl group occupying the S1′ pocket. The strong bidendate hydrogen bond between Arg198 and the amide carbonyl oxygen atom can also be found for this α-alkyl-*N*-aryl mercaptoacetamide subclass (Figure 8B,C).

Concurrently, we have also reported a family of *N*-aryl mercaptosuccinimides (compound **14**) aiming to constrain the flexibility and therefore freeze the active conformation of compound **12**. These inhibitors display activity in the same range as our initial thiol inhibitor **11** (compound **14**, IC_50_ = 3.4 μM vs. compound **11**, IC_50_ = 6.6 μM) [21]. Strikingly, this modification allowed us to reduce the in vivo toxicity in a zebrafish-embryo model: **14** exhibits a maximum tolerated concentration three times higher than **11** (30 μM for **14** vs. 10 μM for **11**). Remarkably, some of our thiol-based inhibitors (compound **13** for example) display good selectivity towards selected human off-targets, including several metalloproteases (MMP-1, MMP-2, MMP-3, MMP-7, MMP-8, MMP-14: <10% inhibition at 100 μM; HDAC-3, HDAC-8: IC_50_ > 100 μM) and excellent cytotoxicity profiles (compound **13**, HepG2, HEK293 and A549 cells: IC_50_ >100 μM). Unfortunately, their further development is limited due to factors such as poor chemical stability (oxidation), a lack of selectivity toward human off-targets or to only moderate activity.

### 3.3. Hydroxamate Compounds

**Figure 9:**
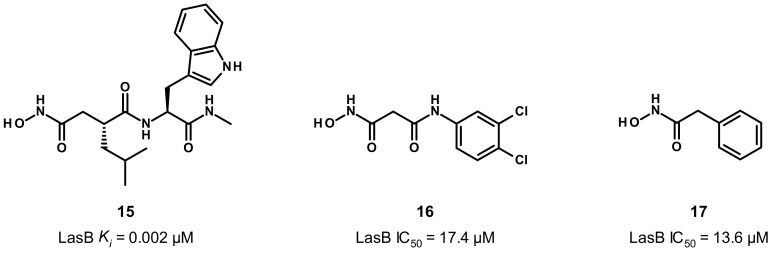
Chemical structures and inhibitory activities of the hydroxamates **15**–**17**. *K_i_* and IC_50_ values were extracted from the original publications [43,45,46].

Given its ability to strongly coordinate the zinc cation, in a bidendate fashion, hydroxamate-based inhibitors were developed (Figure 9). Grobelny et al. have disclosed the inhibition of LasB by hydroxamate dipeptides [43]. The most potent compound in this family was derived from phosphoramidon (**1**) and displays a very strong activity with a low nanomolar *K_i_* value (compound **15**, *K_i_* = 0.002 μM). Although compound **15** was not co-crystallized with LasB, some strong hypotheses on its binding mode can be put forward to explain its high potency, as **15** shows structural similarities to phosphoramidon (**1**) in particular, regarding the side chains in P’_1_ and P’_2_ positions (leucine and tryptophan, respectively). Unfortunately, our docking studies of **15** did not allow us to obtain a presumed coherent binding mode to justify its activity. Moreover, contrary to the thiol, the hydroxamate moiety offers the possibility of a bidendate chelation of the zinc cation, thus giving a rare but documented pentacoordinate geometry [44], which could explain the high potency of **15**.

**Figure 10:**
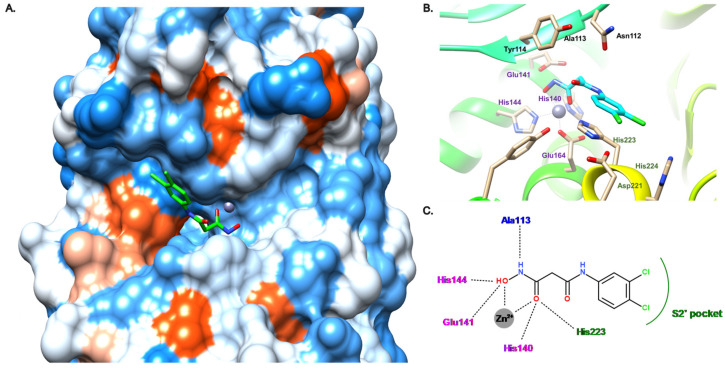
Binding mode of **16** from PDB entry 6FZX [45]. (**A**) View of the active site of LasB, in surface mode, accommodating **16** (green) visualized using Chimera 1.15. The zinc cation is represented as a gray sphere. (**B**) View of the active site of LasB with **16** (blue) and key residues (gray sticks) involved in the binding visualized using Chimera 1.15. (**C**) Two-dimensional schematic representation of the binding mode of **16**. The key interactions are indicated as dashes, the zinc cation is represented as a gray sphere, and the key residues are indicated in pink for those belonging to the S1′ pocket, green for those belonging to the S2′ pocket and blue for the others.

Later, our group described the inhibition of LasB by a malonic compound containing a hydroxamate as the ZBG, aiming to remove the thiol oxidation liability (compound **16**, Figure 9) [45]. However, this thiol replacement by a hydroxamate motif did not allow us to improve the activity, and even resulted in a slight decrease in activity by a factor of 2.6 (compound **16**, IC_50_ = 17.4 μM vs. compound **12**, IC_50_ = 6.6 μM). This drop in activity can be explained by the binding mode of the hydroxamate **16** (Figure 10), which shows differences to inhibitor **12**. As expected, the zinc cation is coordinated by the carbonyl oxygen atom and the hydroxyl of the hydroxamate in a bidentate fashion. The carbonyl oxygen atom forms a hydrogen bond with His223; as a comparison, this histidine residue interacts with the thiol of compound **12**. Additional hydrogen bonds are involved in the binding of **16** such as one from the hydroxyl of the hydroxamate with Glu141 and another between the amide nitrogen and Ala113; both hydrogen bonds do not take part in the binding of **12** in the active site of LasB. Lastly, contrary to **12**, the strong bidentate hydrogen bond involving Arg198 does not participate in the binding of **16**, which can explain the decrease in activity (Figure 10B,C). Moreover, **16** does not occupy the S1′ pocket as the dichlorophenyl group fits in the S2′ pocket (Figure 10A,C). The introduction of a substituent at the malonic position could allow sufficient occupation of the S1′ pocket, and thus significantly increase the potency.

Strikingly, despite its moderate potency and the bad reputation of the hydroxamate motif, the inhibitor **16** displays a good selectivity against other metalloproteases (MMP-1, MMP-2, MMP-3, MMP-7, MMP-8, MMP-14) as well as HDAC-3 and HDAC-8), a low cytoxicity (against HepG2 and HEK293 cells, the effect on HEK293 cells being more pronounced) and good chemical stability profiles, which make it a promising starting point for a medicinal chemistry program. Another family of hydroxamates has been discovered by Janda et al. (compound **17**, IC_50_ = 13.6 μM), but they supposed that these hydroxamates could be unstable as no effect in bacterial LasB assays was observed with them, despite their in vitro micromolar activity [45].

### 3.4. Carboxylate Compounds

**Figure 11:**
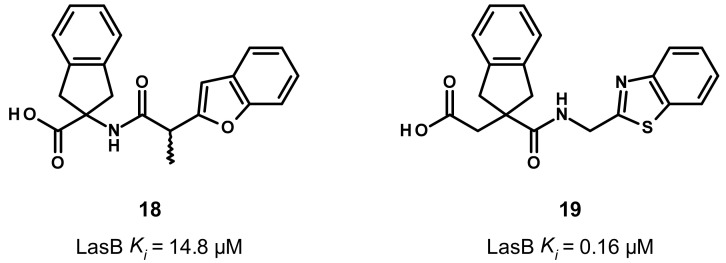
Chemical structures and biochemical activities of the carboxylic acid compounds **18** and **19**. *K_i_* values were extracted from the original publication [47].

Later, the LasB inhibition through small carboxylic acid compounds was disclosed by Leiris et al. (Figure 11) [47]. They initiated a virtual screening based on the structure of LasB and ligand conformation. Regarding their structure-based approach, they have built a pharmacophore containing a ZBG, a hydrophobic moiety to mimic phosphoramidon’s isobutyl chain that fills the S1′ pocket, and at least one hydrogen bond with Glu111, Asn112 or the key Arg198. For their ligand-based approach, they took their inspiration from phosphoramidon. Seven million commercially available compounds were screened using these two complementary approaches. These resulted in the identification of hit compound **18** as a moderate inhibitor of LasB, with a *K*_i_ of 14.8 μM (racemic mixture) and, after SAR-based optimization, **19** as a highly potent inhibitor (*K_i_* = 0.16 μM).

**Figure 12:**
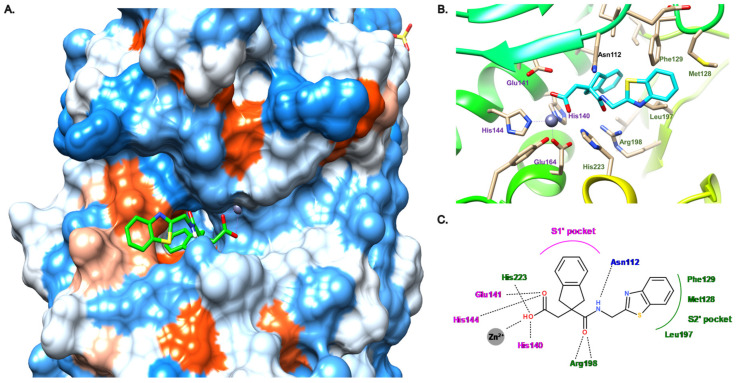
Binding mode of **19** from PDB entry 7AJR [47]. (**A**) View of the active site of LasB, in surface mode, accommodating **19** (green) visualized using Chimera 1.15. The zinc cation is represented as a gray sphere. (**B**) View of the active site of LasB with **19** (blue) and key residues (gray sticks) involved in the binding visualized using Chimera 1.15. (**C**) Two-dimensional schematic representation of the binding mode of **19**. The key interactions are indicated as dashes, the zinc cation is represented as a gray sphere, and the key residues are indicated in pink for those belonging to the S1′ pocket, green for those belonging to the S2′ pocket and blue for the others.

Compound **19** was co-crystallized with LasB, showing a similar binding mode as phosphoramidon (**1**), even though the zinc binding mode is different (Figure 12). Indeed, as expected, the hydroxyl of the carboxylate binds the zinc cation and interacts with His223 through a hydrogen bond, whereas the carbonyl oxygen atom forms a hydrogen bond with Glu141. Moreover, the key Arg198 forms a bidendate hydrogen bond with the amide carbonyl oxygen atom, and the amide nitrogen atom interacts with Asn112 (Figure 12B,C). Interestingly, the indane motif provides a more extensive occupation of the S1′ pocket compared to the isobutyl chain of phosphoramidon, whereas the benzothiazole lies in the S2′ pocket and forms a π–π stacking interaction with Phe129 (Figure 12A,C). This S2′ subsite is occupied by an indole ring in the case of phosphoramidon (**1**) (Figure 3). 

Therefore, compound **19** could be considered as a promising lead for further optimization; however, an enhancement of its potency and a thorough evaluation of its in vitro ADMET parameters as well as its selectivity is still needed.

### 3.5. Other Compounds

**Figure 13:**
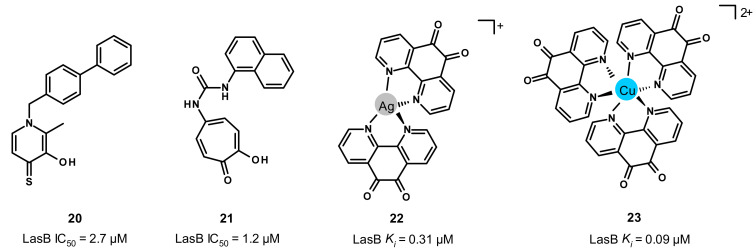
Chemical structures and biochemical activities of compounds **20**–**23**. IC_50_ and *K_i_* values were extracted from the original publications [46,48,49].

Through a screening of a metal chelator library followed by SAR studies, the Cohen and Janda groups reported the inhibition of LasB by 3-hydroxy-2-methyl-4-sulfanylidenepyridin-1(4*H*)-yl (**20**) and tropolone (**21**) with activities in the single-digit micromolar range (IC_50_ = 2.7 and 1.2 μM, respectively) (Figure 13) [46,48]. Unfortunately, our docking studies did not allow us to obtain convincing binding modes of these compounds. Interestingly, despite their limited potencies, compound **20** has a good chemical stability in bacterial culture medium (no detectable S-oxidation or decomposition over a 48 h period), whereas compound **21** exhibits a good selectivity profile (MMP-2, MMP-9, human carbonic anhydrase II and tyrosinase). More recently, Galdino et al. reported that silver and copper complexes of 1,10-phenanthroline-5,6-dione are able to inhibit LasB, in particular, the copper complex **23** with a high potency (*K_i_* = 0.09 μM) (Figure 13) [49]. Although co-crystal structures of these two complexes with LasB are not available at the moment, docking studies were performed by the same group, which enabled us to understand their binding mode and thereby the difference in potency. Regarding the silver-complex **22**, one phenanthroline nitrogen atom forms a hydrogen bond with Ala113, and three water molecules reinforce its binding to the active site by interacting with the carbonyl oxygen and nitrogen atoms of **22**. However, the copper complex **23** does not exhibit the same binding to the active site and interacts with the key residues Arg198 and Asn112, justifying the difference in activity between these two metal complexes. Further investigations about their stability, solubility and selectivity are needed to consider them as drug candidates.

## 4. Conclusions

Since the discovery of phosphoramidon as the first reported inhibitor of LasB, several chemical classes of small molecules have been developed with more or less promising profiles. These compound families allow us to establish a pharmacophore to be considered for the design of future inhibitors: (i) chelation of the zinc cation; (ii) occupation of the S1′ and S2′ binding pockets, S1′ being rather hydrophobic and narrow, and can be considered as the main pocket of interaction, whereas S2′ being more external and wider with a hydrophobic part in the middle and an upper part containing hydrophilic residues, which offers the possibility of hydrogen bonds in particular with Arg198, and to a lesser extent with Asn112 and His223; (iii) accommodation of the small hydrophobic S1′ subpocket close to the zinc cation.

However, none of these inhibitors has reached the market, either due to a lack of potency in vitro or efficacy in vivo, stability or selectivity. Regarding the lack of potency, this issue can be solved thanks to the numerous crystal structures available. Concerning stability and selectivity, replacement of the ZBG could be an interesting opportunity since some functional groups have not been explored yet, such as boronate (vaborbactam) or phosphonate, which have recently shown their ability to strongly inhibit other bacterial collagenases with promising selectivity and stability profiles [22]. Other strategies can be considered to overcome the perpetual challenge of selective metalloprotease inhibition, such as the use of antibodies [50].

Although the antivirulence approach appears to be attractive and promising, other non-antibiotic therapies have also been explored [51], such as the microbiome modulation [52] or the inhibition of the efflux pumps [53,54]. As these approaches will probably require the use of an antibiotic in combination, drug discovery efforts will need to continue in the field of antibiotics as well.

## Data Availability

Data is available on request.
